# Global prevalence of functional dyspepsia according to Rome criteria, 1990–2020: a systematic review and meta-analysis

**DOI:** 10.1038/s41598-024-54716-3

**Published:** 2024-02-20

**Authors:** Kwanjoo Lee, Chang-il Kwon, Abdullah Özgür Yeniova, Ai Koyanagi, Louis Jacob, Lee Smith, Seung Won Lee, Masoud Rahmati, Ju-Young Shin, Jae Il Shin, Wonyoung Cho, Dong Keon Yon

**Affiliations:** 1 Digestive Disease Center, CHA Bundang Medical Center, CHA University School of Medicine, Seongnam, South Korea; 2 Division of Gastroenterology, Department of Internal Medicine, Faculty of Medicine, Tokat Gaziosmanpaşa University, Tokat, Turkey; 3 Research and Development Unit, Parc Sanitari Sant Joan de Deu, Barcelona, Spain; 4 Department of Physical Medicine and Rehabilitation, Lariboisière-Fernand Widal Hospital, AP-HP, Université Paris Cité, Paris, France; 5 Centre for Health, Performance and Wellbeing, Anglia Ruskin University, Cambridge, UK; 6 Department of Precision Medicine, Sungkyunkwan University College of Medicine, Suwon, South Korea; 7 Department of Physical Education and Sport Sciences, Faculty of Literature and Human Sciences, Lorestan University, Khoramabad, Iran; 8 Department of Physical Education and Sport Sciences, Faculty of Literature and Humanities, Vali-E-Asr University of Rafsanjan, Rafsanjan, Iran; 9 School of Pharmacy, Sungkyunkwan University, Suwon, South Korea; 10 Department of Pediatrics, Yonsei University College of Medicine, 50 Yonsei-ro, Seodaemun-gu, Seoul, 03722 South Korea; 11 Center for Digital Health, Medical Science Research Institute, Kyung Hee University Medical Center, Kyung Hee University College of Medicine, 23 Kyungheedae-ro, Dongdaemun-gu, Seoul, 02447 South Korea; 12 Department of Pediatrics, Kyung Hee University College of Medicine, 23 Kyungheedae-ro, Dongdaemun-gu, Seoul, 02447 South Korea

**Keywords:** Dyspepsia, Prevalence, Epidemiology, Functional gastrointestinal disorders, Helicobacter pylori, Gastroenterology, Health care, Medical research

## Abstract

Although functional dyspepsia (FD) is a common functional gastroduodenal disorder with a high socioeconomic burden, little is known about its global prevalence. Thus, we performed a comprehensive study to estimate long-term trends in the prevalence of FD. We searched PubMed/MEDLINE, Embase, and Google Scholar from 1990 to 2022 for population-based studies that reported the prevalence of FD in adults (≥ 18 years old) according to Rome I, II, III, or IV criteria. The prevalence of FD was extracted from included studies to obtain pooled prevalence with 95% confidence intervals (CI) and 95% prediction intervals. Subgroup analysis was performed according to certain characteristics, including geographic region. A total of 44 studies met the eligibility criteria, including 256,915 participants from 40 countries across six continents. The overall global pooled prevalence of FD was 8.4% (95% CI 7.4–.9.5). The prevalence was the highest in Rome I (11.9%; 95% CI 5.1–25.4) and lowest in Rome IV (6.8%; 95% CI 5.8–7.9). Developing countries showed a higher prevalence than developed countries (9.1% versus 8.0%), and prevalence was higher in women, irrespective of the definition used (9.0% versus 7.0%). The pooled prevalence gradually decreased from 1990 to 2020 (12.4% [8.2–18.3] in 1990–2002 versus 7.3% [6.1–8.7] in 2013–2020). The prevalence of FD differs by country, economic status, geographical region, and sex, and the global prevalence has been gradually declining. Despite the heterogeneity of sample population, our study estimates the current global burden of FD and provides information to heath care policy decisions.

## Introduction

Dyspepsia is a complex symptom of gastroduodenal lesions represented by epigastric pain or burning, postprandial fullness, or early satiety. Although many cases are accompanied by an organic pathology that can explain the symptoms, over three-quarters of cases have no organic pathologies^1,2^. Such cases are called functional dyspepsia (FD) and have been defined by the Rome criteria for standardization. Although there are subtle differences depending on the criteria, it is commonly defined as a case where the above symptoms that cannot be explained by investigation including esophagogastroduodenoscopy (EGD) continue for more than 6 months^3^. The prevalence of FD varies depending on the literature and the way it is defined, but is known to be approximately 10–30%^1^. FD is the most common functional gastrointestinal disorder (FGIDs) and is estimated to have the highest healthcare burden^4^. FD can cause social and economic losses by increasing medical expenses and negatively impacting individual mental health^5,6^.

For these reasons, it is necessary to understand the global prevalence of FD, its trend over time, and changes due to the Rome criteria revision to better inform medical policies and determine directions for future research. There have been systematic reviews and meta-analyses on the global prevalence of uninvestigated dyspepsia (UD), indicating patients with dyspepsia who were not received routine investigation and multinational prevalence research conducted by the Rome Foundation based on the Rome IV criteria^2,7–9^. However, no study has comprehensively researched the global prevalence of FD alone based on the time periods of the Rome I to IV (1990 to 2022) criteria. In this study, we estimated the global prevalence of FD diagnosed by the Rome criteria, and also conducted subgroup analysis according to country, Rome criteria, survey method, study year, country level, geographical region, and sex.

## Methods

We performed a systematic review and meta-analysis of relevant studies to investigate global and national trends in the prevalence of FD using the Rome criteria in different countries. Subgroup analysis was done by sex, continent, country level, Rome criteria used for diagnosis, and study year. This systematic review adhered to the Preferred Reporting Items for Systematic Reviews and Meta-Analyses (PRISMA) guidelines^10^ and the protocol was registered with PROSPERO (registration no. CRD42022381641).

### Search strategy and study selection

We searched the PubMed/MEDLINE, Embase, and Google Scholar databases from January 1, 1990, to September 22, 2022, to find studies that reported the incidence or prevalence of FD in adults according to the Rome Foundation diagnostic criteria. We limited the search to 1990 because the Rome criteria were first described in 1991. After excluding pediatric studies and those that did not use the Rome criteria, studies were required to have recruited participants from the general population or have used datasets that were not skewed so could somewhat represent the general population. Those who reported the prevalence of functional dyspepsia in skewed samples, such as university students, certain professions such as military personnel, or a specific sex or age, were excluded. Studies with the same target population or with fewer than 50 participants were also excluded.

The databases were searched using the terms “*functional dyspepsia”* or *“chronic dyspepsia”,* which were combined using the set operator “AND” with studies identified using the terms “*incidence*” *or* “*prevalence*” as free text terms. A total of 2,917 studies were initially selected, and studies that did not meet the selection criteria by title and abstract were excluded. There were no language restrictions, and, if necessary, the contents were verified by translation. A recursive search was performed using the bibliographies of all eligible papers. Two investigators (KL and WC) independently assessed the eligibility of the study and disagreements were resolved through discussion with a third investigator (DKY).

### Data analysis and quality assessment

Data were independently extracted by two investigators (KL and WC) using Microsoft Excel (version 2013; Microsoft, WA, US), with any discrepancies resolved by the opinion of a third investigator (DKY). The following data were collected for each study: study design, country, method of data collection (face-to-face interview, telephone interview, interviewer-administered questionnaire, self-completed postal questionnaire, self-completed questionnaire given to the participant at an appointment, or self-completed Internet-based questionnaire), Rome criteria used to define FD, total number of participants providing complete data, mean age of participants, number of participants with FD, number of male and female participants, and number of male and female participants with FD. When the prevalence of FD was reported according to more than one set of criteria within an individual study, we extracted the participants using the Rome definition. The adapted Newcastle–Ottawa Quality Assessment Scale (NOS)^11^ was used to evaluate the data quality for cross-sectional studies (Tables S1 and S2). This scale has a total score of nine points; high-quality studies have seven or more points, medium-quality ones have four to six points, and low-quality studies have three points or less.

A random effects model was used to calculate the pooled prevalence of FD. We performed extensive subgroup analyses stratified by country, sex, study year (1990–2002, 2003–2012, and 2013–2020), country income level (developed and developing countries), geographical region by continent (Africa, Asia, Australia, Europe, North America, and South America), Rome criteria used to define FD, and source of participant data (interview- and non-interview-based data). Countries were divided into developed and developing countries based on the UN Human Development Index (cut-off point 0.8). Based on the results of the subgroup analysis by study year. The global prevalence of FD was compared using DerSimonian and Laird (DL) pooled estimates^12^ with 95% confidence intervals (CIs). To reduce the error rate, the Hartung-Knapp-Sidik-Jonkman (HKSJ) method was also used in the analysis^13^. Heterogeneity was assessed using the I^2^ statistic^14^ and Egger’s test was performed for publication bias. The prediction interval (95% PI) was used to make our main results for the summary of estimates more robust in order to assess the uncertainty of our findings using Bayesian statistics^15^. Microsoft Excel (version 2013; Microsoft, WA, US) and R software (version 4.1.2; R Foundation, Vienna, Austria) were used to calculate the main results and generate all tables. A two-sided *p* value of < 0.05, was considered significant^16–25^.

### Patient and public involvement

No patients were involved in setting the research question or outcome measures, nor were they involved in developing plans for the design or implementation of the study. None of the patients were asked to advise on the interpretation or writing of the results. However, we plan to disseminate the results of this study to all study participants or wider relevant communities on request.

## Results

**Figure 1:**
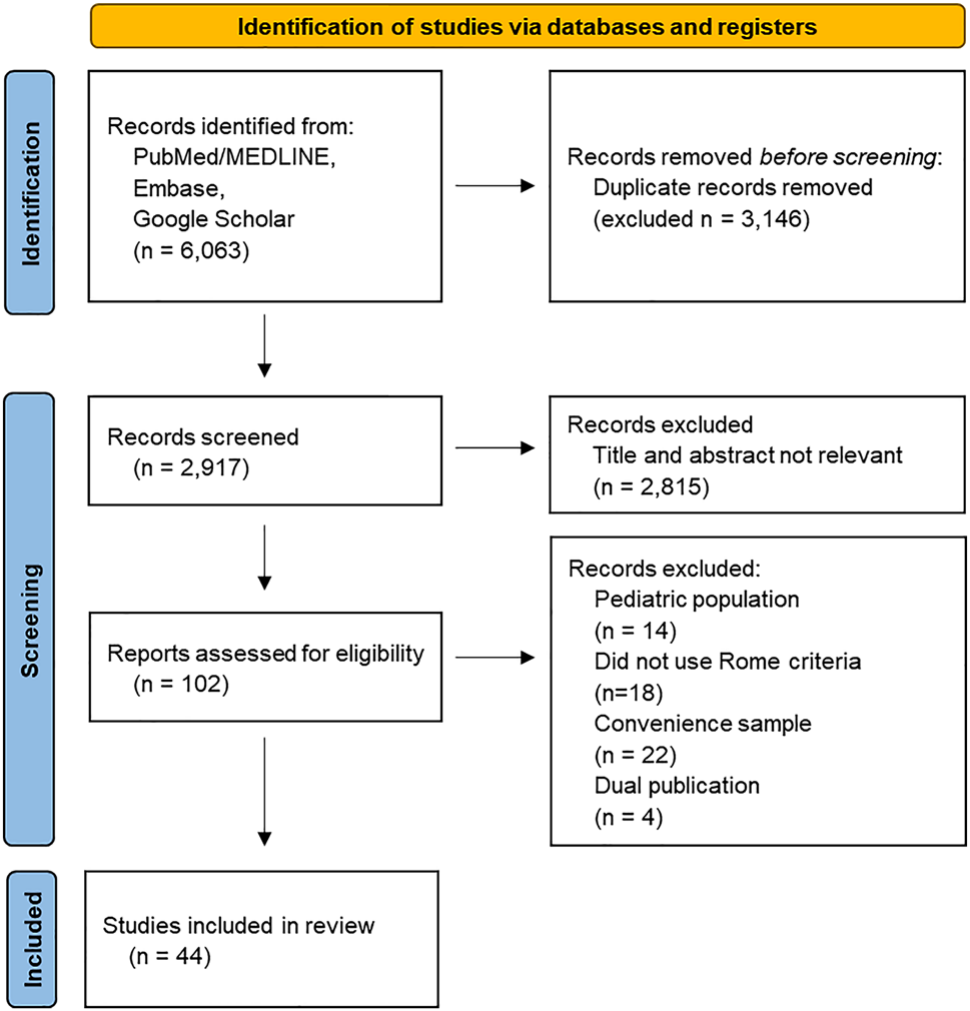
Literature search and study selection.

**Figure 2:**
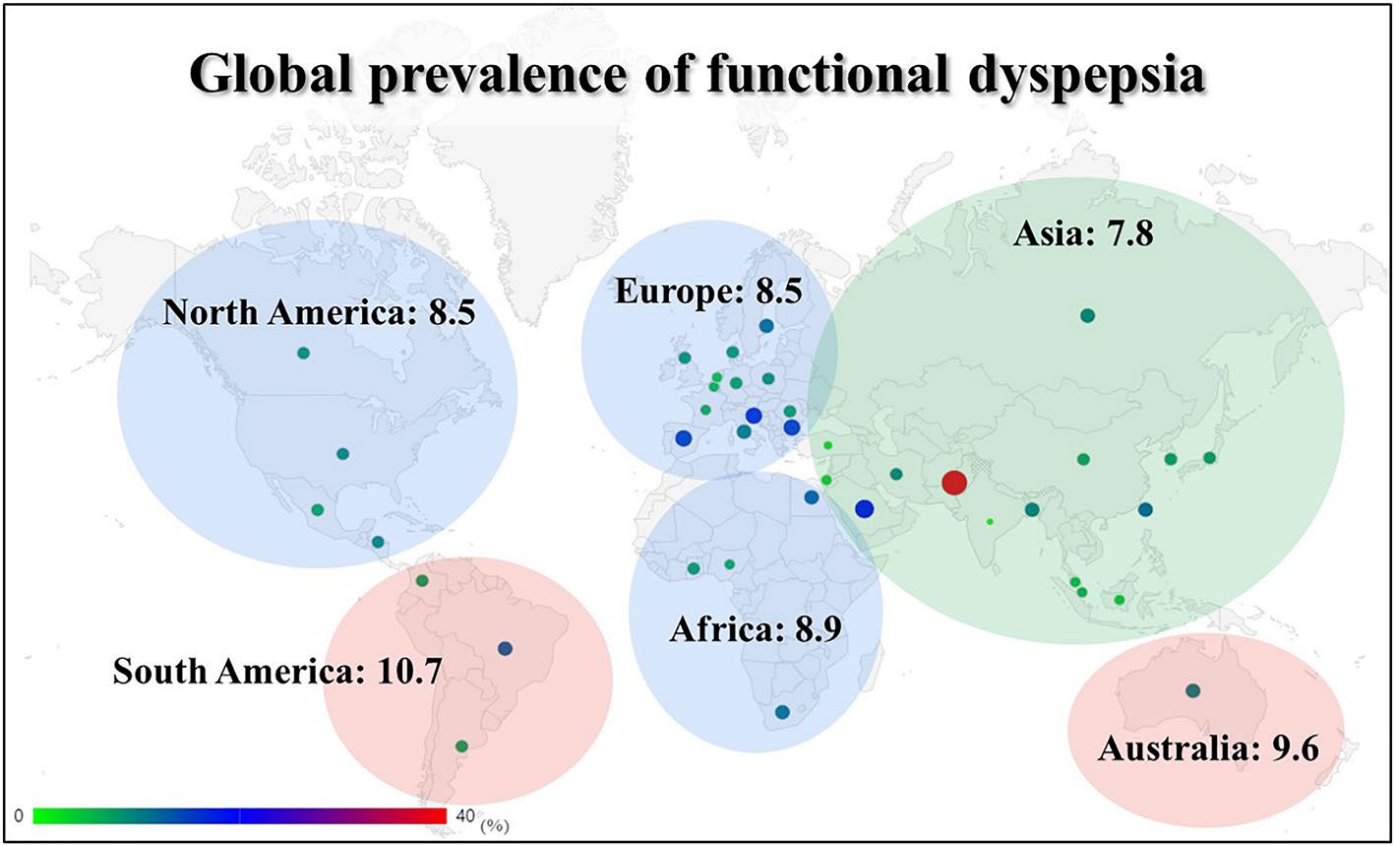
A graphical abstract of the prevalence of functional dyspepsia by continent.

The search strategy identified 6,063 studies. Of these, 44 studies met the eligibility criteria^8,9,26–67^, representing 80 separate adult study populations, comprising 256,915 participants from 40 countries worldwide (Figs. 1, 2 and Table 1). Most studies were conducted in a single country, with the exception of a three-nation study conducted in Canada, the UK, and the USA^8^ and a multinational survey conducted in 33 different countries^9^. FD data of patients from 1993 to 2020 were included; most were cross-sectional studies, but several population-based cohort studies, case–control studies, and randomized control studies that could calculate the prevalence were also included. Most of the study population was a randomly sampled general population; however, samples from health check-up visitors, general hospital visitors (not patients), and large-scale hospital staff were also included. Face-to-face interviews and internet-based self-completed questionnaires accounted for the majority of the survey methods, but there were also some methods using self-completed postal questionnaires, telephone interviews, or self-completed questionnaires at appointments. The detailed characteristics of all included studies are shown in Table S1.

**Table 1:** Prevalence of functional dyspepsia.

	Number of populations	Number of participants	Pooled prevalence, % (95% CI)	Prevalence by HKSJ method, % (95% CI)	Heterogeneity $I^{2}$, %	Egger’s P value	95% *p*redicted interval
Overall	80	256,915	8.4 (7.4–9.5)	9.9 (8.5–11.3)	98.833	0.481	2.6–23.8
Defining criteria							
Rome I	3	10,278	11.9 (5.1–25.4)	13.6 (0.0–37.8)	99.525	0.806	0.0–100.0
Rome II	5	5,742	10.6 (6.1–17.7)	12.4 (2.7–22.0)	98.048	0.070	1.1–55.2
Rome III	27	150,923	10.8 (8.7–13.4)	12.5 (9.4–15.6)	99.352	0.184	3.1–31.7
Rome IV	45	89,972	6.8 (5.8–7.9)	7.9 (6.6–9.1)	97.635	0.308	2.3–18.4
By survey type							
Interview based†							
Rome I	–	–	–	–	–	–	–
Rome II	3	3492	12.1 (5.3–25.3)	13.7 (0.0–35.8)	98.644	0.087	0.0–100.0
Rome III	16	51,402	10.1 (7.7–13.2)	12.1 (7.3–16.8)	98.860	0.894	2.9–29.5
Rome IV	11	20,766	4.4 (2.5–7.7)	6.1 (2.6–9.6)	98.765	0.000	0.4–33.0
Non-interview†							
Rome I	3	10,278	11.9 (5.1–25.4)	13.6 (0.0–37.8)	99.525	0.806	0.0–100.0
Rome II	2	2250	8.6 (2.2–27.8)	10.3*	98.236	–	–
Rome III	11	99,521	12.0 (8.1–17.3)	13.1 (9.0–17.2)	99.607	0.223	2.4–43.1
Rome IV	34	69,206	7.8 (6.7–8.9)	8.4 (7.1–9.6)	96.799	0.000	3.2–17.6
By study year							
1990–2002	8	15,578	12.4 (8.2–18.3)	14.0 (7.8–20.2)	98.794	0.668	2.5–43.6
2003–2012	15	56,840	10.1 (7.8–13.1)	11.3 (8.0–14.5)	99.033	0.388	3.1–28.2
2013–2020	53	132,931	7.3 (6.1–8.7)	8.8 (7.1–10.5)	98.566	0.747	1.9–23.8
By country level							
Developed country	53	183,426	8.0 (6.8–9.3)	10.0 (8.3–11.7)	98.850	0.787	2.4–23.4
Developing country	27	73,489	9.1 (7.4–11.2)	12.1 (9.5–14.8)	98.703	0.231	2.8–26.0
By continent							
Africa	4	6673	8.9 (6.5–12.0)	9.2 (4.5–13.9)	93.802	0.026	1.9–32.9
Asia	36	96,152	7.8 (6.1–9.8)	10.5 (7.5–13.4)	99.174	0.003	1.7–28.9
Australia	6	11,536	9.6 (7.1–12.9)	10.3 (6.0–14.6)	96.546	0.140	3.0–26.6
Europe	21	115,469	8.5 (7.1–10.2)	9.1 (7.4–10.9)	98.207	0.130	3.4–19.6
North America	9	22,325	8.5 (7.4–9.7)	8.7 (7.3–10.5)	89.680	0.826	5.1–13.8
South America	4	4760	10.7 (7.8–14.6)	11.3 (3.9–18.6)	90.734	0.470	2.3–38.1

*CI* confidence interval, *HKSJ* Hartung-Knapp-Sidik-Jonkman.

*In the HKSJ method, the confidence interval of prevalence is unavailable when the number of studies is less than three.

^†^The interview-based type refers to face-to-face interviews, interviewer-administered questionnaires, telephone interviews, and non-interview types refer to other methods.

The global pooled prevalence using the DL method of FD in the 80 included adult population was 8.4% (95% confidence interval [CI] 7.4–9.5; I^2^ = 98.8%), and the global prevalence using the HKSJ method was 9.9% (95% CI 8.5–11.3), with a 95% PI of 2.6–23.8 (Table 1). The lowest prevalence of FD reported was 0.7% (95% CI 0.5–1.0) in an Indian population cross-sectional study that administered the Rome IV questionnaire during face-to-face household interviews. The highest prevalence was 38.3% (95% CI 35.1–41.6), which was reported in a Pakistani population cross-sectional study that used the Rome III criteria in face-to-face interviews (Table 2).

**Table 2:** Prevalence of functional dyspepsia by country.

Country, k = 40	Rome overall, % (95% CI)	Rome I criteria, % (95% CI)	Rome II criteria, % (95% CI)	Rome III criteria, % (95% CI)	Rome IV criteria, % (95% CI)
Argentina	6.9 (5.9–8.1)	–	–	–	6.9 (5.9–8.1)
Australia	10.2 (7.4–13.8)	8.5 (7.5–9.5)	4.3 (3.1–6.0)	14.0 (12.7–15.4)	7.2 (6.2–8.4)
Bangladesh	10.5 (5.3–19.8)	–	–	7.6 (6.3–9.1)	19.4 (17.7–21.2)
Belgium	5.0 (4.1–6.0)	–	–	–	5.0 (4.1–6.0)
Brazil	12.3 (8.9–16.6)	–	–	13.6 (7.3–23.9)	10.6 (9.3–12.0)
Bulgaria	15.3 (10.6–21.5)	–	–	–	15.3 (10.6–21.5)
Canada	8.1 (7.3–9.0)	–	–	–	8.1 (7.3–9.0)
China	7.6 (3.2–17.1)	–	23.5 (21.0–26.2)	5.1 (4.4–6.0)	5.1 (3.7–6.9)
Colombia	7.2 (6.2–8.4)	–	–	–	7.2 (6.2–8.4)
Croatia	16.6 (13.9–19.6)	–	–	16.6 (13.9–19.6)	–
Denmark	7.8 (7.6–8.0)	–	–	7.8 (7.6–8.0)	–
Egypt	12.3 (10.9–13.8)	–	–	–	12.3 (10.9–13.8)
France	5.8 (2.7–12.0)	–	–	4.0 (3.8–4.2)	8.5 (7.4–9.8)
Germany	6.9 (5.9–8.1)	–	–	–	6.9 (5.9–8.1)
Ghana	7.2 (5.9–8.8)	–	–	–	7.2 (5.9–8.8)
Honduras	9.4 (7.6–11.7)	–	–	–	9.4 (7.6–11.7)
India	0.7 (0.5–1.0)	–	–	–	0.7 (0.5–1.0)
Indonesia	4.4 (3.4–5.7)	–	–	–	4.4 (3.4–5.7)
Iran	9.8 (7.0–13.4)	–	–	12.6 (9.4–16.7)	2.9 (2.2–3.8)
Israel	3.6 (2.8–4.5)	–	–	–	3.6 (2.8–4.5)
Italy	9.9 (8.2–12.0)	–	11.0 (9.3–13.1)	–	9.1 (7.9–10.4)
Japan	8.1 (4.7–21.0)	–	–	11.9 (4.7–26.8)	2.4 (1.9–3.1)
Malaysia	5.0 (2.2–11.1)	–	–	–	5.0 (2.2–11.1)
Mexico	6.6 (5.6–7.8)	–	–	–	6.6 (5.6–7.8)
Netherlands	4.1 (3.3–5.0)	–	–	–	4.1 (3.3–5.0)
Nigeria	6.0 (4.9–7.4)	–	–	–	6.0 (4.9–7.4)
Pakistan	38.3 (35.1–41.6)	–	–	38.3 (35.1–41.6)	–
Poland	8.3 (7.2–9.6)	–	–	–	8.3 (7.2–9.6)
Romania	7.4 (6.4–8.6)	–	–	–	7.4 (6.4–8.6)
Russia	10.3 (9.0–11.7)	–	–	–	10.3 (9.0–11.7)
Saudi Arabia	18.9 (17.5–20.4)	–	–	–	18.9 (17.5–20.4)
Singapore	5.9 (5.0–7.0)	–	–	–	5.9 (5.0–7.0)
South Africa	11.0 (9.7–12.4)	–	–	–	11.0 (9.7–12.4)
South Korea	7.9 (4.2–14.5)	–	6.3 (5.2–7.7)	10.0 (3.6–25.1)	4.9 (4.0–5.9)
Spain	15.3 (13.2–17.6)	–	–	–	15.3 (13.2–17.6)
Sweden	11.4 (5.9–20.9)	–	–	15.7 (13.6–18.1)	8.2 (7.1–9.5)
Taiwan	12.6 (4.5–30.7)	24.8 (22.9–26.8)	16.1 (14.3–18.0)	4.5 (3.9–5.2)	–
Turkey	2.4 (0.5–11.1)	–	–	–	2.4 (0.5–11.1)
UK	7.9 (6.6–9.7)	–	–	–	7.9 (6.6–9.7)
USA	8.9 (7.2–10.9)	7.4 (6.8–8.1)	–	9.8 (8.8–10.8)	9.2 (6.4–13.0)

*CI* confidential interval.

### Prevalence of FD according to Rome criteria and survey year

In total, three studies stated that they used the Rome I criteria in three separate countries, five used the Rome II criteria in five separate countries, 27 used the Rome III criteria in 14 separate countries, and 45 used the Rome IV criteria in 38 separate countries. The pooled prevalence of FD was highest according to Rome I criteria and lowest according to Rome IV criteria (11.9% [95% CI 5.1–25.4] vs. 6.8% [95% CI 5.8–7.9], respectively). The prevalence using the HKSJ method also had the highest frequency when using Rome I criteria and the lowest frequency when Rome IV criteria was used (13.6% [95% CI 0.0–37.8] vs. 7.9% [95% CI 6.6–9.1], respectively). Table 2 summarizes the pooled data for the prevalence of FD worldwide according to country, using all iterations of the Rome criteria.

**Figure 3:**
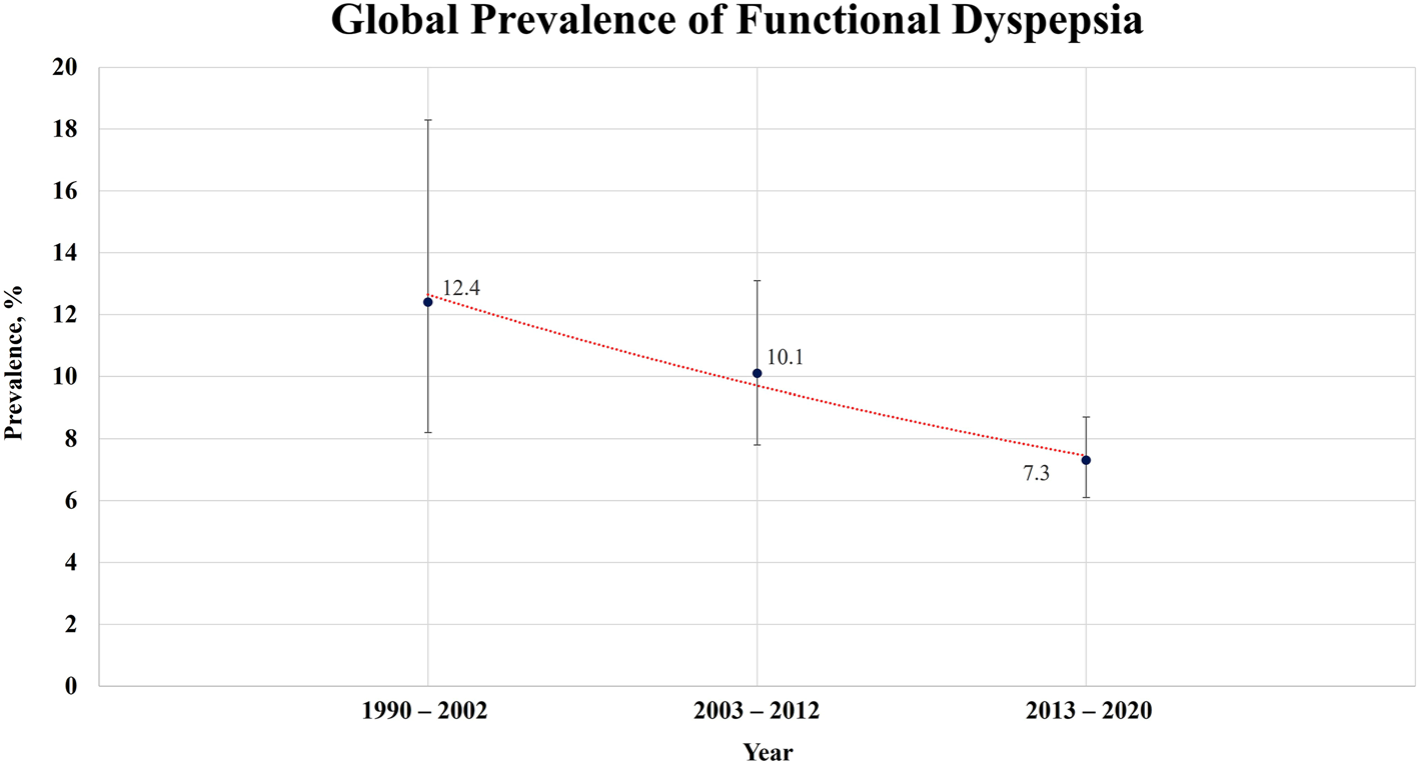
Global prevalence of functional dyspepsia from 1990 to 2020.

There was a total of 41 studies whose survey year was specified in the research methods: eight between 1990 and 2002, 15 between 2003 and 2012, and 53 between 2013 and 2020. The pooled prevalence was the highest between 1990 and 2002 and the lowest between 2013 and 2020 (12.4% [95% CI 8.2–18.3] vs. 7.3% [95% CI 6.1–8.7], respectively), and the prevalence using the HKSJ method showed similar results (Table 1). The prevalence of FD tended to decrease over time (Fig. 3).

### Prevalence of FD according to country income level and continent

53 out of the 80 surveys were conducted in developed countries, and the remaining 37 were conducted in developing countries. The prevalence in developed countries was 8.0% (95% CI 6.8–9.3) using the DL method and 10.0% (95% CI 8.3–11.7) using the HKSJ method. In developing countries, it was 9.1% (95% CI 7.4–11.2) and 12.1% (95% CI 9.5%–14.8%), respectively, showing a higher trend in developing countries (Table 1).

When the included study population was classified by continent, Asia had the largest number of studies (36 studies), followed by Africa and South America (4 studies). In both DL and HKSJ methods, the prevalence was highest in South America at 10.7% (95% CI 7.8–14.6) and 11.3% (95% CI 3.9–18.6), respectively, but the continent with the lowest prevalence was Asia with DL method (7.8%, 95% CI 7.8–14.6) and with the HKSJ method, it was North America (8.7%, 95% CI 7.3–10.5; Table 1 and Fig. 2).

### Prevalence of FD according to survey method

We analyzed several survey methods by classifying them into two categories. These were the interview-type in which a trained interviewer participates in the survey in any way, and a non-interview type in which a questionnaire is prepared and submitted by the participant. For each Rome criterion used in the definition, the prevalence of the two types of survey methods was compared, but in the case of Rome I, there was no interview-type study, so a comparison could not be made. In Rome II, the interview-type showed a higher pooled prevalence (12.1% [95% CI 5.3–25.3] vs. 8.6% [95% CI 2.2–27.8], respectively) than the non-interview type, and showed a similar trend in the HKSJ method. However, in Rome III and Rome IV, which accounted for the majority of included studies, the interview-type tended to be significantly lower than the non-interview type (10.1% [95% CI 7.7–13.2] vs. 12.0% [95% CI 8.1–17.3], and 4.4% [95% CI 2.5–7.7] vs. 7.8% [95% CI 6.7–8.9]).

### Prevalence of FD according to sex

The prevalence of FD was divided into male and female categories and analyzed by dividing the Rome criteria into Rome overall, Rome I, II, III, and IV (Table 3). The prevalence of women in all categories was significantly higher, and the largest difference was observed in Rome IV with an odds ratio for female to male of 2.68 (95% CI 2.31–3.12).

**Table 3:** Prevalence of functional dyspepsia for each of the Rome criteria by sex.

	Number of studies	Proportion of females with functional dyspepsia, %	Proportion of males with functional dyspepsia, %	Odds ratio for females to males, % (95% CI)	*p*-value
Rome overall	32	9.0	7.0	**1.32 (1.27–1.37)**	** < 0.001**
Rome I	3	12.3	9.8	**1.29 (1.14–1.46)**	** < 0.001**
Rome II	5	14.0	11.0	**1.31 (1.13–1.54)**	** < 0.001**
Rome III	21	8.2	6.3	**1.33 (1.27–1.39)**	** < 0.001**
Rome IV	3	24.3	10.7	**2.68 (2.31–3.12)**	** < 0.001**

*CI* confidential interval.

The bold numbers indicate a significant difference (*p* < 0.05).

### Study quality assessment

A total of 44 studies included in the analysis were evaluated for quality using the NOS scale. Except for one study that showed poor selection quality, all other studies showed good or fair selection and outcomes quality. In the comparability section, 81.8% (36/44) of the studies were of good or fair quality (Table S2). Twenty-six studies were evaluated as high-quality, and the remaining 18 studies were evaluated as medium-quality. None of the included studies was of low quality.

## Discussion

### Key findings

This systematic review and meta-analysis integrated data from 44 original articles and over 256,915 participants from 40 countries across six continents (Africa, Asia, Australia, Europe, North America, and South America), which reported the prevalence of FD in the community using all iterations of the Rome criteria from 1990 to 2020. We investigated whether the prevalence of FD could vary according to country, continent, and sex, even if the same definition was applied. The estimated global prevalence of FD was 8.4% (95% CI 7.4–9.5). This study also found differences in prevalence between developed and developing countries, with a higher prevalence in developing countries. The prevalence according to the Rome criteria definitions showed a change of less than 1.5% between Rome I, II, and III but showed a sharp decrease in the prevalence with the Rome IV criteria. Regardless of the definition used, we found that the prevalence of FD was significantly higher in women than that in men. Finally, according to study year, we found that the worldwide prevalence of FD showed gradual decreased.

### Plausible mechanism

The prevalence of FD showed a gradual decrease by the study year, which can be attributed to the following reasons. First, there is a decrease in the prevalence of *Helicobacter pylori* (*H. pylori*) infection, which is assumed to be a cause of FD^68–70^. For various reasons, such as the prevention of gastric cancer, *H. pylori* eradication is widely practiced, and as a result, the prevalence of *H. pylori* infection worldwide is decreasing and this result was seen in a systematic review of global prevalence; hence, the prevalence of FD is also estimated to have decreased^69–71^. This claim is supported by the fact that *H. pylori* prevalence is high in developing countries and low in developed countries^68^. *H. pylori* infection without peptic ulcers or gastric cancer may be associated with FD, and there is substantial evidence that eradication improves symptoms in FD patients with H. pylori infection^70^.

Second, it could be because the diagnostic accuracy for structural disease has increased with the development of various technologies and the evaluation techniques for gastroesophageal non-structural organic causes, such as motility disorder^72–74^. The third explanation is a reduction in infectious gastroenteritis (IGE). It is a well-known fact that the incidence of FD increases after suffering from IGE^75,76^. It can be assumed that the decrease in the incidence of IGE along with the improvement of global sanitation contributed to the decrease in the prevalence of FD, and the difference in prevalence between developed and developing countries in our study supports this rationale^77^. Lastly, the Rome criteria for FD diagnosis has become increasingly stringent as classifications have become more diverse^78^. Especially, in Rome III, postprandial distress syndrome, one of the subtypes of FD, could be identified even if symptoms occurred just once a week, but in Rome IV criteria, the requirements were made more stringent to three times a week^79^. This is supported by the reduced prevalence using the Rome IV criteria compared to Rome I, II, and III.

When the odds ratio for prevalence by sex was analyzed, women consistently showed higher odds for every Rome criteria as well as overall prevalence, compared with men. This trend agrees with the other global prevalence studies of functional constipation (FC), irritable bowel syndrome (IBS), and other FGIDs using the Rome criteria^80,81^. This consistent trend indicates that differences between males and females due to various causes affect the disease prevalence. Visceral hypersensitivity is known to have a significant effect on sex differences in animal models; however, it has not been clearly identified in humans^76^. Other possible causes include gastric emptying and colon transit time, the degree of sensation for visceral pain due to structural differences in hormones or the brain, brain response to stress, ghrelin, heredity, low-grade inflammation of the duodenum, and food; however, a clear cause has not yet been identified^76,82^.

### Strengths and limitations

This study has several limitations. First, few studies have been conducted in Africa or South America, and more studies have been conducted in developed countries than in developing countries. Second, there are more studies using Romes III and IV criteria than those using Romes I and II. Third, there was variability in the data collection methods, such as face-to-face or telephone interviews versus self-completed internet-based or paper questionnaires, which may have caused differences in ascertaining the prevalence of FD. In fact, when subgroup analysis was performed by dividing the survey types in this study, a difference in prevalence was observed despite the Rome criteria used for the definition being the same. Fourth, although an atypical population was excluded, some study populations were not pure general population. However, these studies had sufficient sample sizes and included patients of different ages. Fifth, because the subtype definition of FD was different in Rome I, II, and Rome III and IV, the prevalence was not investigated separately for each subtype. Sixth, demographic data such as sex, age, or socioeconomic status could not be obtained from all studies, and only sex was available for subgroup analysis^83,84^ Seventh, all the studies included in the analysis used a means to exclude organic causes and defined the participants as having FD accordingly, but few studies performed routine EGD, and most studies were health check-up population studies. In some studies, ultrasound or CT of the abdomen was performed in addition to EGD. Therefore, the prevalence of true FD in the study populations may be questionable^85^. However, it is practically impossible to accurately distinguish FD from organic dyspepsia by any process, and each country has a different approach to the investigation of dyspepsia^1,86,87^. Even if investigations, including EGD, were not conducted in the study, it cannot be said that participants were not received investigation. In addition, it is difficult to conduct research that routinely performs invasive tests such as EGD on the general population^88^. Therefore, in many studies, even if it was not a routine investigation, after using a method to exclude organic dyspepsia, FD is defined by the Rome criteria and the prevalence is investigated. This is also the case in the large-scale multinational study conducted by the Rome Foundation^8,9^. Lastly, there was considerable heterogeneity. Given that our analyses involved heterogeneity, it's important to note that some results may be subject to inaccuracy. Therefore, interpretation should be approached with caution, considering these aspects. To the best of our knowledge, our study is the first to investigate the global prevalence of FD, including the prevalence based on Rome I to IV criteria. And we performed an extensive literature search and sophisticated statistics, including the DL method, HKSJ method, and 95% PI, to reduce the error rate and obtain more accurate results.

### Further considerations

As mentioned above, the decrease in the prevalence of FD can be assumed to be due to the development of various medical examination techniques and a decrease in the morbidity of *H. pylori* and IGE. However, the cause of FD remains unclear, as mentioned in the discussion on sex differences. Therefore, efforts to find a clear cause for FD are still needed. Reason analysis and institutional efforts are needed to reduce the frequency of FD in developing countries as much as in developed countries. In addition, despite global cross-sectional studies, prevalence studies in Africa and South America are still insufficient^9^. For an accurate comparison, additional epidemiological studies are needed in these countries in the future.

Although the same criteria were used in our study, there was a slight difference in prevalence according to the survey method, which is similar to studies on other FGIDs using the Rome criteria^80,81^. This fact suggests that although the meaning of the Rome criteria is sufficient as a global standard, the rate at which patients report symptoms may vary depending on the survey method; therefore, standardization of the survey method or a process for correcting the prevalence according to the survey method is needed. These aspects should be considered when revising the Rome criteria in the future.

## Conclusions

This systematic review and meta-analysis determined the global prevalence of FD to range from 11.9%, as per the Rome I criteria, to 6.8% based on the Rome IV criteria spanning the years 1990 to 2020. Despite heterogeneity across studies, our analysis unveiled noteworthy insights into the global prevalence of FD. Notably, there were variations between developed and developing countries, with a higher prevalence observed in developing nations. Additionally, our findings consistently demonstrated a significantly higher prevalence of FD in women compared to men, irrespective of the diagnostic criteria employed. This study provides crucial estimates of the current global burden of FD, offering valuable insights for future healthcare planning and research priorities.

### Supplementary Information

Supplementary Information.

## Data Availability

All data are provided in the Article and in the appendix.
