# Supplementary material for: Global prevalence of functional dyspepsia according to Rome criteria, 1990–2020: a systematic review and meta-analysis
Source: Sci Rep. 2024 Feb 20;14:4172. doi: 10.1038/s41598-024-54716-3 (PMC10879214; doi:10.1038/s41598-024-54716-3)

| **Supplement Material** |
| --- |

Original Article

**Global prevalence of functional dyspepsia according to Rome criteria, 1990-2020: A systematic review and meta-analysis**

Running head: **Global prevalence of functional dyspepsia**

Kwanjoo Lee^1^, Chang-il Kwon^1^, Abdullah Özgür Yeniova^2^, Ai Koyanagi^3^, Louis Jacob^3,4^, Lee Smith^5^, Seung Won Lee^6^, Masoud Rahmati^7,8^, Ju-Young Shin^9^, Jae Il Shin^10*^, Wonyoung Cho^11*^, Dong Keon Yon^11,12*^

***Corresponding authors**

**Table S1.** Summary of included studies.

| **First author, year** | **Country** | **Number of**  **subjects, n** | **Rome criteria** | **Study design** | **Survey type** | **Mean age, year (SD)** |
| --- | --- | --- | --- | --- | --- | --- |
| Abid et al, 2022 | Pakistan | 860 | Rome III | Cross-sectional | Face-to-face interview | 37.2 (11.4) |
| Adibi et al, 2016 | Iran | 4,763 | Rome III | Cross-sectional | Interviewer-administered questionnaire | 36.9 (8.1) |
| Almeida et al, 2017 | Brazil | 548 | Rome III | Cross-sectional | Self-completed questionnaire at appointment | 36.0 (16.1) |
| Aro et al, 2009 | Sweden | 1,001 | Rome III | Cross-sectional | Face-to-face interview | 54.1 (-) |
| Aziz et al, 2018 | Canada | 1,988 | Rome IV | Cross-sectional | Self-completed internet-based questionnaire | - |
| Aziz et al, 2018 | UK | 1,994 | Rome IV | Cross-sectional | Self-completed internet-based questionnaire | - |
| Aziz et al, 2018 | USA | 1,949 | Rome IV | Cross-sectional | Self-completed internet-based questionnaire | - |
| Beh et al, 2021 | Malaysia | 1,002 | Rome III | Cross-sectional | Face-to-face interview | 33.8 (10.9) |
| Boyce et al, 2006 | Australia | 762 | Rome II | Cross-sectional | Self-completed postal questionnaire | 47.0 (13.7) |
| Chang et al, 2012 | Taiwan | 4,275 | Rome III | Cross-sectional | Face-to-face interview | 52.4 (2.4) |
| Choung et al, 2012 | USA | 3,517 | Rome III | Population-based cohort | Self-completed postal questionnaire | 61.0 (15.0) |
| Chuah et al, 2021 | Malaysia | 1,002 | Rome IV | Cross-sectional | Face-to-face interview | 33.8 (10.9) |
| Drossman et al, 1993 | USA | 5,430 | Rome I | Cross-sectional | Self-completed postal questionnaire | 49.1 (15.9) |
| Ebling et al, 2016 | Croatia | 663 | Rome III | Cross-sectional | Self-completed postal questionnaire | 43.6 (-) |
| Hu et al, 2021 | China | 2,916 | Rome III | Randomized controlled trial | Face-to-face interview | 56.9 (6.9) |
| Jung et al, 2016 | South Korea | 363 | Rome III | Case-control | Telephone interview | 50.0 (10.9) |
| Kaji et al, 2010 | Japan | 2,680 | Rome III | Cross-sectional | Self-completed questionnaire at appointment | 39.5 (13.7) |
| Kim et al, 2018 | South Korea | 1,714 | Rome III | Cross-sectional | Interviewer-administered questionnaire | 51.5 (12.7) |
| Koloski et al, 2002 | Australia | 2,910 | Rome I | Cross-sectional | Self-completed postal questionnaire | 43.8 (14.5) |
| Koloski et al, 2015 | Australia | 767 | Rome III | Cross-sectional | Self-completed postal questionnaire | 59.9 (11.5) |
| Koloski et al, 2016 | Australia | 1,900 | Rome III | Population-based cohort | Self-completed questionnaire at appointment | 57.0 (14.0) |
| Koloski et al, 2020 | Australia | 3,161 | Rome III | Cross-sectional | Self-completed questionnaire at appointment | 54.3 (15.6) |
| Kovács et al, 2022 | UK | 888 | Rome IV | Cross-sectional | self-completed internet-based questionnaire | 43.9 (18-89)* |
| Le Pluart et al, 2015 | France | 35,447 | Rome III | Population-based cohort | self-completed internet-based questionnaire | 49.7 (14.3) |
| Lee et al, 2009 | South Korea | 1,443 | Rome II | Cross-sectional | Face-to-face interview | 48.6 (14.9) |
| Lee et al, 2013 | Malaysia | 160 | Rome III | Cross-sectional | Interviewer-administered questionnaire | 39.6 (14.3) |
| Li et al, 2002 | China | 1,016 | Rome II | Cross-sectional | Face-to-face interview | - |
| Lima et al, 2022 | Brazil | 201 | Rome III | Case-control | Self-completed questionnaire at appointment | 55.5 (12.8) |
| Lu et al, 2005 | Taiwan | 1,938 | Rome I | Cross-sectional | Self-completed questionnaire at appointment | 54.7 (11.4) |
| Lu et al, 2005 | Taiwan | 1,488 | Rome II | Cross-sectional | Self-completed questionnaire at appointment | 55.0 (11.3) |
| Miwa et al, 2012 | Japan | 3,547 | Rome III | Cross-sectional | Self-completed internet-based questionnaire | - |
| Nakov et al, 2020 | Bulgaria | 1,896 | Rome IV | Cross-sectional | Self-completed internet-based questionnaire | 35.5 (11.7) |
| Nakov et al, 2022 | Bulgaria | 980 | Rome IV | Cross-sectional | Self-completed internet-based questionnaire | 40.5 (-) |
| Noh et al, 2010 | South Korea | 2,388 | Rome III | Cross-sectional | Interviewer-administered questionnaire | 43.2 (8.4) |
| Norwood et al, 2021 | Honduras | 815 | Rome IV | Cross-sectional | Face-to-face interview | 40.6 (17.0) |
| Perveen et al, 2014 | Bangladesh | 3,000 | Rome III | Observational | Face-to-face interview | 33.9 (16.4) |
| Rahman et al, 2021 | Bangladesh | 3,351 | Rome III | Cross-sectional | Face-to-face interview | 40.4 (16.1) |
| Rasmussen et al, 2015 | Denmark | 47,090 | Rome III | Population-based cohort | Self-completed postal questionnaire | 52.0 (12.0) |
| Seyedmirzaei et al, 2014 | Iran | 2,210 | Rome III | Cross-sectional | Face-to-face interview | 43.4 (16.3) |
| Sharbafchi et al, 2020 | Iran | 4,763 | Rome III | Cross-sectional | Interviewer-administered questionnaire | 36.5 (7.9) |
| Sorouri et al, 2010 | Iran | 18,180 | Rome III | Cross-sectional | Face-to-face interview | 38.7 (0.3) |
| Sperber et al, 2021 | Argentina | 2,057 | Rome IV | Cross-sectional | Self-completed internet-based questionnaire | 47.6 (17.2) |
| Sperber et al, 2021 | Australia | 2,036 | Rome IV | Cross-sectional | Self-completed internet-based questionnaire | 47.4 (17.2) |
| Sperber et al, 2021 | Bangladesh | 2,018 | Rome IV | Cross-sectional | Face-to-face interview | 47.5 (17.2) |
| Sperber et al, 2021 | Belgium | 2,021 | Rome IV | Cross-sectional | Self-completed internet-based questionnaire | 47.3 (17.2) |
| Sperber et al, 2021 | Brazil | 2,004 | Rome IV | Cross-sectional | Self-completed internet-based questionnaire | 47.5 (17.2) |
| Sperber et al, 2021 | Canada | 2,029 | Rome IV | Cross-sectional | Self-completed internet-based questionnaire | 47.4 (17.2) |
| Sperber et al, 2021 | China | 2,710 | Rome IV | Cross-sectional | Face-to-face interview | 48.8 (16.4) |
| Sperber et al, 2021 | China | 2,914 | Rome IV | Cross-sectional | Self-completed internet-based questionnaire | 47.3 (17.2) |
| Sperber et al, 2021 | Colombia | 2,007 | Rome IV | Cross-sectional | Self-completed internet-based questionnaire | 46.1 (16.3) |
| Sperber et al, 2021 | Egypt | 2,020 | Rome IV | Cross-sectional | Self-completed internet-based questionnaire | - |
| Sperber et al, 2021 | France | 2,019 | Rome IV | Cross-sectional | Self-completed internet-based questionnaire | 47.1 (17.0) |
| Sperber et al, 2021 | Germany | 2,020 | Rome IV | Cross-sectional | Self-completed internet-based questionnaire | 47.2 (17.1) |
| Sperber et al, 2021 | Ghana | 1,190 | Rome IV | Cross-sectional | Face-to-face interview | 46.9 (17.0) |
| Sperber et al, 2021 | India | 4,592 | Rome IV | Cross-sectional | Face-to-face interview | 46.0 (16.6) |
| Sperber et al, 2021 | Indonesia | 1,231 | Rome IV | Cross-sectional | Face-to-face interview | 47.6 (17.1) |
| Sperber et al, 2021 | Iran | 1,840 | Rome IV | Cross-sectional | Face-to-face interview | 47.3 (17.2) |
| Sperber et al, 2021 | Italy | 2,063 | Rome IV | Cross-sectional | Self-completed internet-based questionnaire | 47.4 (17.2) |
| Sperber et al, 2021 | Japan | 2,504 | Rome IV | Cross-sectional | Self-completed internet-based questionnaire | 47.2 (17.2) |
| Sperber et al, 2021 | Malaysia | 1,976 | Rome IV | Cross-sectional | Face-to-face interview | 51.0 (15.4) |
| Sperber et al, 2021 | Mexico | 2,001 | Rome IV | Cross-sectional | Self-completed internet-based questionnaire | 47.1 (17.1) |
| Sperber et al, 2021 | Netherlands | 2,008 | Rome IV | Cross-sectional | Self-completed internet-based questionnaire | 47.5 (17.2) |
| Sperber et al, 2021 | Nigeria | 1,442 | Rome IV | Cross-sectional | Face-to-face interview | 47.2 (16.9) |
| Sperber et al, 2021 | Poland | 2,057 | Rome IV | Cross-sectional | Self-completed internet-based questionnaire | 47.3 (17.2) |
| Sperber et al, 2021 | Romania | 2,049 | Rome IV | Cross-sectional | Self-completed internet-based questionnaire | 43.7 (13.6) |
| Sperber et al, 2021 | Russia | 2,000 | Rome IV | Cross-sectional | Self-completed internet-based questionnaire | 47.3 (17.1) |
| Sperber et al, 2021 | Singapore | 2,047 | Rome IV | Cross-sectional | Self-completed internet-based questionnaire | 43.7 (13.6) |
| Sperber et al, 2021 | South Africa | 2,021 | Rome IV | Cross-sectional | Self-completed internet-based questionnaire | 41.2 (15.5) |
| Sperber et al, 2021 | South Korea | 2,022 | Rome IV | Cross-sectional | Self-completed internet-based questionnaire | 45.5 (14.9) |
| Sperber et al, 2021 | Spain | 2,072 | Rome IV | Cross-sectional | Self-completed internet-based questionnaire | 47.4 (17.2) |
| Sperber et al, 2021 | Sweden | 2,084 | Rome IV | Cross-sectional | Self-completed internet-based questionnaire | 47.5 (17.2) |
| Sperber et al, 2021 | Turkey | 1,950 | Rome IV | Cross-sectional | Face-to-face interview | 40.3 (14.7) |
| Sperber et al, 2021 | Turkey | 2,010 | Rome IV | Cross-sectional | Self-completed internet-based questionnaire | 41.7 (14.9) |
| Sperber et al, 2021 | USA | 2,023 | Rome IV | Cross-sectional | Self-completed internet-based questionnaire | 47.5 (17.2) |
| Sperber et al, 2021 | UK | 2,027 | Rome IV | Cross-sectional | Self-completed internet-based questionnaire | 47.5 (17.2) |
| Sperber et al, 2022 | Israel | 2,012 | Rome IV | Cross-sectional | Self-completed internet-based questionnaire | 44.6 (16.4) |
| Tanaka et al, 2019 | Japan | 456 | Rome III | Cross-sectional | Interviewer-administered questionnaire | 50.0 (41-59)† |
| Zacharakis et al, 2020 | Saudi Arabia | 2,811 | Rome IV | Cross-sectional | Self-completed internet-based questionnaire | 25.0 (18-58)† |
| Zagari et al, 2010 | Italy | 1,033 | Rome II | Cross-sectional | Face-to-face interview | 58.7 (-) |
| Zand Irani et al, 2021 | USA | 2,573 | Rome IV | Cross-sectional | Self-completed postal questionnaire | 28.8 (6.2) |

Abbreviations: SD, standard deviation

*Mean age and year range are presented.

†Median and interquartile ranges are provided.

**Table S2.** Quality assessment of each included study using the Newcastle-Ottawa Scale.

|  | **Selection†** | | | | **Comparability††** | **Outcomes†††** | | | **Total** | | |
| --- | --- | --- | --- | --- | --- | --- | --- | --- | --- | --- | --- |
| **Author, year** | **S1** | **S2** | **S3** | **S4** | **C1** | **O1** | **O2** | **O3** | **Selection** | **Comparability** | **Outcomes** |
| Abid et al, 2022 | * | * | * | - | * | * | * | * | Good | Fair | Good |
| Adibi et al, 2016 | * | * | * | - | ** | * | * | * | Good | Good | Good |
| Almeida et al, 2017 | * | * | - | - | - | * | * | - | Fair | Poor | Good |
| Aro et al, 2009 | * | * | - | - | ** | * | * | * | Fair | Good | Good |
| Aziz et al, 2018 | * | * | - | - | * | * | * | * | Fair | Fair | Good |
| Beh et al, 2021 | - | * | * | - | ** | * | * | * | Fair | Good | Good |
| Chang et al, 2012 | * | * | * | - | ** | * | * | * | Good | Good | Good |
| Choung et al, 2012 | * | * | - | * | * | * | * | - | Good | Fair | Good |
| Chuah et al, 2021 | - | * | * | - | - | * | * | * | Fair | Poor | Good |
| Drossman et al, 1993 | * | * | - | * | ** | * | * | * | Good | Good | Good |
| Ebling et al, 2016 | * | * | - | - | ** | * | * | * | Fair | Good | Good |
| Hu et al, 2021 | - | * | * | * | ** | * | * | * | Good | Good | Good |
| Jung et al, 2016 | * | * | - | * | ** | * | * | - | Good | Good | Good |
| Kaji et al, 2010 | * | * | - | - | ** | * | * | * | Fair | Good | Good |
| Kim et al, 2018 | * | * | * | - | ** | * | * | * | Good | Good | Good |
| Koloski et al, 2015 | * | * | - | * | ** | * | * | * | Good | Good | Good |
| Koloski et al, 2016 | * | * | - | * | -- | * | - | * | Good | Poor | Good |
| Koloski et al, 2020 | * | * | - | - | -- | * | * | * | Fair | Poor | Good |
| Kovács et al, 2022 | * | * | - | - | ** | * | * | * | Fair | Good | Good |
| Le Pluart et al, 2015 | * | * | - | * | * | * | * | - | Good | Fair | Good |
| Lee et al, 2009 | * | * | * | - | ** | * | * | - | Good | Good | Good |
| Lee et al, 2013 | * | * | * | - | ** | * | * | * | Good | Good | Good |
| Li et al, 2002 | * | * | * | - | ** | * | * | * | Good | Good | Good |
| Lima et al, 2022 | - | * | - | - | * | * | * | * | Poor | Fair | Good |
| Lu et al, 2005 | * | * | * | - | ** | * | * | * | Good | Good | Good |
| Miwa et al, 2012 | * | * | - | - | -- | * | * | * | Fair | Poor | Good |
| Nakov et al, 2020 | * | * | - | - | ** | * | * | * | Fair | Good | Good |
| Nakov et al, 2022 | * | * | - | - | -- | * | * | * | Fair | Poor | Good |
| Noh et al, 2010 | * | * | - | - | -- | * | * | * | Fair | Poor | Good |
| Norwood et al, 2021 | * | * | * | - | -- | * | * | * | Good | Poor | Good |
| Perveen et al, 2014 | * | * | * | - | ** | * | * | * | Good | Good | Good |
| Rahman et al, 2021 | * | * | * | - | * | * | * | * | Good | Fair | Good |
| Rasmussen et al, 2015 | * | * | - | * | ** | * | * | - | Good | Good | Good |
| Seyedmirzaei et al, 2014 | * | * | * | - | ** | * | * | * | Good | Good | Good |
| Sharbafchi et al, 2020 | * | * | - | - | * | * | * | * | Fair | Fair | Good |
| Sorouri et al, 2010 | * | * | * | - | * | * | * | * | Good | Fair | Good |
| Sperber et al, 2022 | * | * | - | - | ** | * | * | * | Fair | Good | Good |
| Tanaka et al, 2019 | * | * | - | - | ** | * | * | * | Fair | Good | Good |
| Zacharakis et al, 2020 | * | * | - | - | ** | * | - | * | Fair | Good | Good |
| Zagari et al, 2010 | * | * | * | - | * | * | * | * | Good | Fair | Good |
| Zand Irani et al, 2021 | * | * | - | - | * | * | * | * | Fair | Fair | Good |

† Selection: Poor (0–1), fair (2), and good (3–4).
 In cohort studies

S1. Representativeness of the exposed cohort (1)
 S2. Selection of the non-exposed cohort (1)
 S3. Ascertainment of exposure (1)
 S4. Demonstration that the outcome of interest was not present at the start of the study (1)

In cross-sectional studies

S1. Representativeness of the target population (1)
 S2. Sample size (1)
 S3. Ascertainment of exposure (1)
 S4. Non-respondent (1)

†† Comparability: Poor (0), fair (1), or good (2).
 C1. Comparability of cohorts or target populations based on design or analysis (2)

††† Outcome: Poor (0–1), Good (2–3)

In cohort studies
 O1. Assessment of outcome
 O2. Follow-up was long enough for outcomes to occur
 with O3. Adequacy of follow-up of cohorts

In cross-sectional studies

O1~2. Assessment of outcome
 O3. Statistical test

**Figure S1.** Global prevalence of functional dyspepsia by Rome criteria


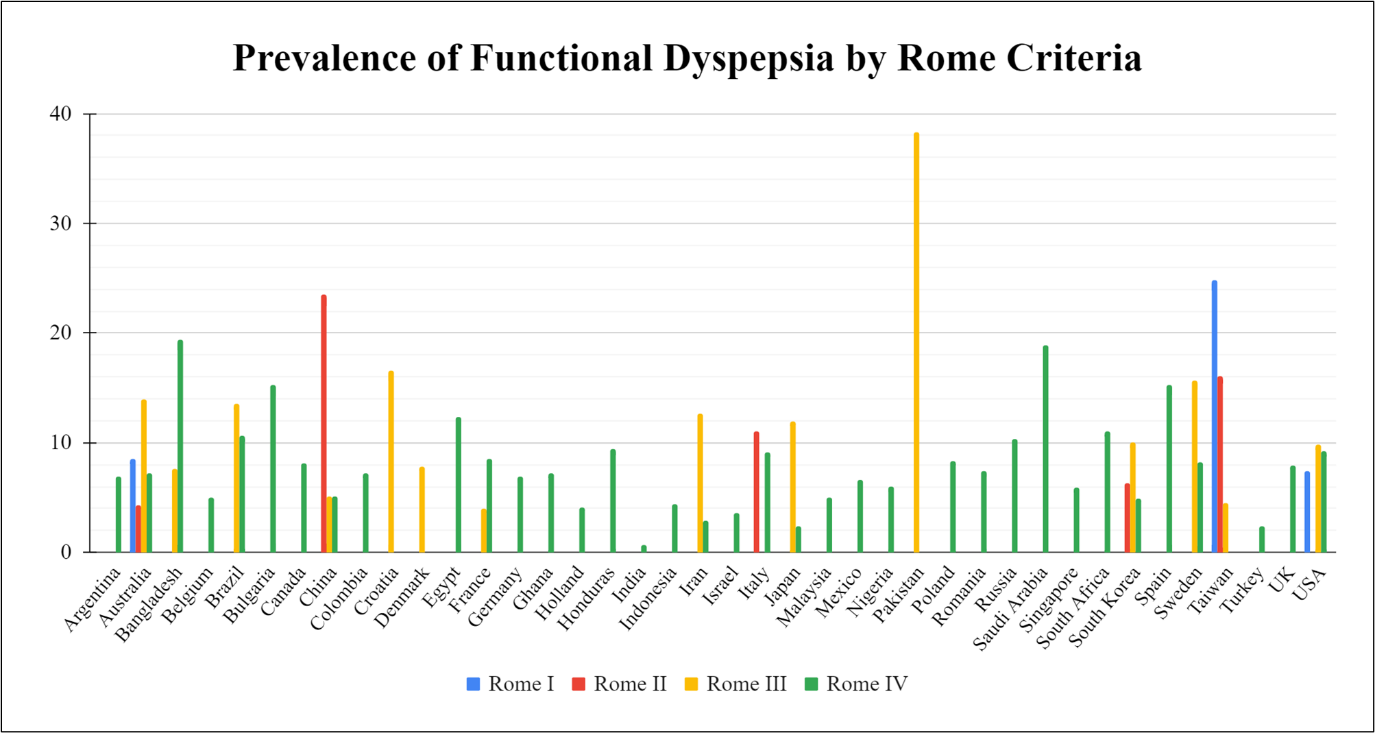

Supplement: Supplementary file 1 — Supplementary Information. [file 41598_2024_54716_MOESM1_ESM.docx]
